# Understanding the Impacts of Land-Use Policies on a Threatened Species: Is There a Future for the Bornean Orang-utan?

**DOI:** 10.1371/journal.pone.0049142

**Published:** 2012-11-07

**Authors:** Serge A. Wich, David Gaveau, Nicola Abram, Marc Ancrenaz, Alessandro Baccini, Stephen Brend, Lisa Curran, Roberto A. Delgado, Andi Erman, Gabriella M. Fredriksson, Benoit Goossens, Simon J. Husson, Isabelle Lackman, Andrew J. Marshall, Anita Naomi, Elis Molidena, Nardiyono, Anton Nurcahyo, Kisar Odom, Adventus Panda, Purnomo, Andjar Rafiastanto, Dessy Ratnasari, Adi H. Santana, Imam Sapari, Carel P. van Schaik, Jamartin Sihite, Stephanie Spehar, Eddy Santoso, Amat Suyoko, Albertus Tiju, Graham Usher, Sri Suci Utami Atmoko, Erik P. Willems, Erik Meijaard

**Affiliations:** 1 Research Centre in Evolutionary Anthropology and Palaeoecology, School of Natural Sciences and Psychology, Liverpool John Moores University, Liverpool, United Kingdom; 2 Centre for International Forestry Research, Situ Gede, Bogor Barat, West Java, Indonesia; 3 Durrell Institute for Conservation and Ecology, School of Anthropology and Conservation, University of Kent, Canterbury, United Kingdom; 4 Sabah Wildlife Department, Kota Kinabalu, Sabah, Malaysia; 5 HUTAN, Kinabatangan Orang-utan Conservation Programme, Kota Kinabalu, Sabah, Malaysia; 6 North England Zoological Society, Chester Zoo, Chester, United Kingdom; 7 The Woods Hole Research Center, Falmouth, Massachusetts, United States of America; 8 Orangutan Foundation, London, United Kingdom; 9 Woods Institute for the Environment, Stanford University, California, United States of America; 10 Department of Anthropology, Stanford University, Stanford, California, United States of America; 11 Departments of Anthropology and Biological Sciences, Program in Integrative and Evolutionary Biology (IEB), University of Southern California, Los Angeles, United States of America; 12 GFA/KWF, Kapuas Hulu Program, West Kalimantan, Indonesia; 13 Sumatran Orangutan Conservation Programme (PanEco-YEL), Medan, Sumatra, Indonesia; 14 Institute for Biodiversity and Ecosystem Dynamics/Zoological Museum, University of Amsterdam, Amsterdam, The Netherlands; 15 Danau Girang Field Centre, c/o Sabah Willife Department, Kota Kinabalu, Sabah, Malaysia; 16 Organisms and Environment Division, Cardiff School of Biosciences, Cardiff University, Cardiff, United Kingdom; 17 Orangutan Tropical Peatland Project, c/o the Center for International Cooperation in the Sustainable Management of Tropical Peatlands (CIMTROP), University of Palangka Raya, Central Kalimantan, Indonesia; 18 Department of Anthropology, Graduate Group in Ecology, Animal Behavior Graduate Group, University of California Davis, California, United States of America; 19 Forum Orangutan Indonesia (FORINA), Bogor, West Java, Indonesia; 20 The Nature Conservancy (TNC), Jakarta, Indonesia; 21 Borneo Orangutan Survival Foundation (BOSF), Nyaru Menteng, Central Kalimantan, Indonesia; 22 World Wide Fund for Nature-Indonesia (WWF-Indonesia), Sebangau Conservation Program/University of Palangkaraya, Central Kalimantan, Indonesia; 23 Flora and Fauna International-Indonesia, Ragunan, Jakarta, Indonesia; 24 Lembaga Living Landscapes Indonesia (LLI), Pontianak, West Kalimantan, Indonesia; 25 Biology Faculty, Universitas Nasional (UNAS), Jakarta, Indonesia; 26 Yayasan Orangutan Indonesia (YAYORIN), Pangkalan Bun, Central Kalimantan, Indonesia; 27 Anthropological Institute and Museum, University of Zurich, Zurich, Switzerland; 28 Restorasi Habitat Orangutan Indonesia (RHOI), Bogor, West Java, Indonesia; 29 Anthropology Program, Department of Religious Studies and Anthropology, University of Wisconsin Oshkosh, Oshkosh, Wisconsin, United States of America; 30 World Wide Fund for Nature-Indonesia (WWF-Indonesia), West Kalimantan Program, Indonesia; 31 People and Nature Consulting International, Ciputat, Jakarta, Indonesia; 32 School of Biological Sciences, University of Queensland, Brisbane, Australia; 33 School of Archaeology and Anthropology, Australian National University, Canberra, Australia; Texas A & M University, United States of America

## Abstract

The geographic distribution of Bornean orang-utans and its overlap with existing land-use categories (protected areas, logging and plantation concessions) is a necessary foundation to prioritize conservation planning. Based on an extensive orang-utan survey dataset and a number of environmental variables, we modelled an orang-utan distribution map. The modelled orang-utan distribution map covers 155,106 km^2^ (21% of Borneo's landmass) and reveals four distinct distribution areas. The most important environmental predictors are annual rainfall and land cover. The overlap of the orang-utan distribution with land-use categories reveals that only 22% of the distribution lies in protected areas, but that 29% lies in natural forest concessions. A further 19% and 6% occurs in largely undeveloped oil palm and tree plantation concessions, respectively. The remaining 24% of the orang-utan distribution range occurs outside of protected areas and outside of concessions. An estimated 49% of the orang-utan distribution will be lost if all forest outside of protected areas and logging concessions is lost. To avoid this potential decline plantation development in orang-utan habitats must be halted because it infringes on national laws of species protection. Further growth of the plantation sector should be achieved through increasing yields in existing plantations and expansion of new plantations into areas that have already been deforested. To reach this goal a large scale island-wide land-use masterplan is needed that clarifies which possible land uses and managements are allowed in the landscape and provides new standardized strategic conservation policies. Such a process should make much better use of non-market values of ecosystem services of forests such as water provision, flood control, carbon sequestration, and sources of livelihood for rural communities. Presently land use planning is more driven by vested interests and direct and immediate economic gains, rather than by approaches that take into consideration social equity and environmental sustainability.

## Introduction

Orang-utans are heralded as global conservation icons [1]. This international status may provide the necessary leverage to influence land-use decisions and effective strategic policies to conserve this threatened species in the wild. The orang-utan (*Pongo* spp) is classified as endangered [2]. The main threats to the species are habitat loss, fragmentation, and hunting [3], [4], [5], [6], [7], [8], [9]. Lowland forests (<500 m a.s.l) are the primary habitat for orang-utans, but these forests have been increasingly logged or converted to high-revenue industrial plantations (oil palm, rubber and timber), and small-scale agriculture [e.g. [10], [11], [12]]. These activities and increasing road networks in these lowland habitats have resulted in a large number of orang-utans killed each year [6], [9], [13]. Hunting for orang-utans is widespread on Borneo, with about half of the cases occurring in areas within or in close proximity to agri-and silvi-cultural plantations, as well as in natural forest areas [7].

In an effort to safeguard orang-utans (*Pongo* spp.) the Indonesian government pledged in 2007 to stabilize all remaining wild populations of orang-utan by the year 2017 [14]. The Malaysian state of Sabah has issued similar goals in its recent orang-utan action plan [15]. To achieve these aims, the Indonesian and Malaysian governments must acquire up-to-date knowledge of the current geographic distribution of orang-utans. In Sabah, the distribution of orang-utans is already well-known [16] and this information has lead to a 30-year logging ban in key orang-utan habitat, and facilitated initiatives such as the Bio-Bank biodiversity offsetting project [17]. Yet, accurate knowledge of the orang-utan distribution in Indonesia and the Malaysian state of Sarawak remains inadequate.

In this study, we focus on the Bornean orang-utan (*Pongo pygmaeus*), a species which has been relatively well studied, but lacks the comprehensive spatial management plans that are needed to fulfil national and state level commitments from the Indonesian and Malaysian governments. The last comprehensive update on the distribution of Bornean orang-utans in the wild was based on orang-utan location/presence data from the 1990 s and the early 2000 s [5]. These data were not geo-referenced, and it was assumed that orang-utans distribution was only constrained by altitude, and only occurred in lowland forests (<500 m asl), without considering other ecological, climatic or anthropogenic factors important in predicting the species' current geographic extent. Since year 2000, a large number of new surveys have been conducted throughout Borneo using Global Positioning Systems. These new geo-referenced locations of orang-utan presence provide a more complete and updated account of current orang-utan distribution within forests and the rapidly developed multi-use production landscapes in Borneo.

Here, we present a new distribution map for the Bornean orang-utan based on a predictive modelling approach [e.g.[18]]. The last decade has seen an upsurge of models to predict species potential (i.e. areas of suitability) and actual distributions [for an overview see [19], [20]]. We use MaxEnt, a maximum-entropy model [21], [22]. This model was chosen because it performs well with presence-only data [20].

The current paper has three aims: 1) to provide an update of the orang-utan distribution across Borneo; 2) to assess which spatial factors influence this distribution; and 3) to determine how much of the orang-utan distribution occurs in protected areas, and in areas allocated for the expansion of a) industrial oil palm plantations, b) industrial tree plantations, and c) logging concessions.

## Methods

### Species occurrence samples

Species presence surveys were carried out between 1990 and 2011 by twenty-four researchers throughout the island of Borneo. A total of 6,711 presence point locations (mostly from nest sightings) were collected using a GPS, and compiled into a GIS database. During a workshop on the 8^th^ and 9^th^ of December 2011 attended by seven orang-utan specialists with differing regional knowledge of orang-utan populations and collectively over 100 years of working with orang-utans in Borneo, the location points were divided into four categories: 1) rare sightings that were either due to very low density in the area or transient orang-utans; 2) common sightings in areas with multiple sightings and in which orang-utans are assumed or known to be common; 3) data points in areas where most forest had disappeared during the past two decades and for which it was not known whether orang-utans still persisted; and, 4) areas from which orang-utans had been rescued (mostly forests cleared for plantation development in Central Kalimantan Province). Allocation to these categories was based on field experience as well as information gathered during interview surveys in 2008 and 2009 [7]. Due to inevitable sample bias, the location points were not evenly distributed across the landscape. The points were clustered in areas that had been surveyed more intensively than others. To minimize spatial autocorrelation in these clusters, we employed a 10 km^2^ fishnet grid over the samples and randomly selected one point from each grid. We used the Hawth Tools extension (http://www.spatialecology.com/htools/tooldesc.php) in ArcGis 9.3.1 for the preparation of the grid and the random selection procedure. After filtering, a total of 558 points remained, and we used these location points of orang-utan presence to generate the species distribution model. All fieldwork was conducted with permits from the necessary authorities in Indonesia and Malaysia.

### Contextual layers

The contextual layers included maps of climatic, topographic, soil, above ground carbon stock [23], land cover and road density. The contextual layers (see Table 1) were prepared at a 30 arc-second (approximately 1×1 km) resolution for the model and were selected based on knowledge of factors that might be important in determining orang-utan distributions. Although we realize that hunting has an impact on orang-utan density and distribution [7], [8], [9], [24], [25] there is no hunting intensity layer available and therefore this variable was not used. Several of these layers were downloaded from open web-sources and re-sampled to our model's extent whilst other layers needed processing. A digital elevation model from the National Aeronautics Space Administration's Shuttle Radar Topography Mission (NASA SRTM) was used to generate elevation and slope maps [26]; a rugosity layer was generated from the DEM using the DEM surface tools [27]. To generate a road index map, we digitized logging roads and main roads using Landsat TM images from 2000 (obtained from: www.usgs.gov). From these data, we developed a road density layer using the “line density” tool in ArcGIS 9.3.1. Road density was expressed as km/km^2^. A 5 km radius was drawn around each 1×1 km raster cell and the length of each road line that fell within the circle was measured. These lengths were then summed (if more than one line fell in the circle) and divided by the surface area of the 5 km radius circle.

**Table 1:** Contextual layers used for the generation of the orang-utan distribution.

Layer	Source
Annual rainfall	http://www.worldclim.org/
Mean daily temperature	http://www.worldclim.org/
Mean daily temperature range	http://www.worldclim.org/
Yearly variation in rainfall	http://www.worldclim.org/
Elevation (DEM)	http://srtm.csi.cgiar.org/
Slope	Generated from elevation DEM
Rugosity	Generated from elevation DEM
Soil types	http://www.iiasa.ac.at/Research/LUC/External- World-soil-database/HTML/HWSD_Data.html?sb = 4
Above-ground carbon stock	From [23]
Land cover	http://www.eorc.jaxa.jp/SAFE/LC_MAP/
Road density	Digitized from Landsat images

Note: Layer correlations (Using ENMTools [84]) were lower than 0.5 except for elevation with slope and rugosity. All three layers were maintained in the final analyses since they are of different importance for orang-utans.

### MaxEnt Model

To generate the model we used the auto feature settings within the MaxEnt software [http://www.cs.princeton.edu/~schapire/maxent/see [22]]. The model output is a map with a cumulative probability distribution output ranging from 0 to 1; with 0 meaning absent and 1 meaning the highest probability of presence. To generate a binary presence-absence map from the raw MaxEnt output, we determined a cut-off probability threshold. Below this threshold, orang-utans are considered absent and above it, they are considered present. A variety of thresholds were evaluated, yet for the final orang-utan distribution map a probability threshold of 0.15 was set because this value maximized the sensitivity of the model, and minimized the predicted area while remaining realistic based on our current field-based understanding of where orang-utans occur. A 10-fold cross-validation model was run to evaluate the errors around the fitted function and the models predictive performance. The range of values can be used to assess the stability of the model. We also assessed variable importance by using the jackknife procedure function in MaxEnt. This procedure creates a model including: (1) all variables; (2) a model for each of the variables in isolation; and, (3) a model excluding each variable, but including all the remaining variables. Because the aim of this study was to produce an operational map, one that can be used for spatial prioritisation and inform land-use decision making, we wanted to go beyond the limitations of model validation techniques when true-absence data is lacking and where the dataset has sample bias as these issues can obscure true model performance in producing a realistic distribution map. To undertake a post-model critique and review the final orang-utan distribution map, we held the above-mentioned 2-day workshop. This workshop resulted in the omitting of some areas from the predicted orang-utan distribution map; where it was known that orang-utans do not occur at present (e.g. most of southeast Kalimantan). Additionally, the workshop also resulted in the inclusion of other areas where orang-utans are known to exist but were not present in the MaxEnt output, for example within Kutai National Park, and the adjacent industrial monoculture timber plantations in East Kalimantan [28].

### Protected areas and production landscapes

To determine how much of the orang-utan distribution occurs in protected areas, and in areas allocated for the expansion of: a) industrial oil palm plantations, b) industrial tree plantation, and c) logging concessions, we compiled information on protected areas and concessions across Borneo. Due to unavailability of data on mining concessions, these were not included in the analyses.

#### Protected areas

Despite being legally prohibited deforestation and logging occur in many protected areas [e.g. [29]]. These include national parks, nature, wildlife sanctuaries and game reserves, recreational parks, virgin jungle reserves, and protection forests. Maps of existing protected areas in Borneo were compiled from various government sources. For the four Indonesian provinces of West, Central, South and East Kalimantan, protected area boundaries were obtained from provincial spatial plans (*Rencana Tata Ruang Wilayah Propinsi*) or from national spatial plans (*Paduserasi*) at a scale of 1∶250,000, or from National Park offices at 1∶50,000 scale wherever such local boundary delineation was available (Gunung Palung and Danau Sentarum National Parks). For the Malaysian province of Sabah, protected area boundaries were obtained from Sabah's Forestry Department, at a scale of 1∶250,000. For the Malaysia province of Sarawak and for Brunei, protected area boundaries were obtained from the World Database of Protected Areas [30].

#### Industrial oil palm plantation (IOPP) concessions

Concessions for industrial oil palm plantations (IOPP) are granted by governments to allow the establishment and management of industrial, monoculture oil palm estates. In Indonesia, since decentralisation in the year 2000 [31], the majority of new IOPP permits have been issued by district level governments on lands categorised as ‘conversion forests’ by national land use plans. ‘Conversion forests’ (HPK *Hutan Produksi Konversi*, and APL *Areal Penggunaan Lain*) include regions allocated explicitly for non-forest purposes. A smaller fraction of the permits have been issued on lands classified as ‘production forests’, in areas where there is a deficit of ‘conversion forests’, for example in the province of Central Kalimantan. Similar procedures are followed by the state governments of Sabah and Sarawak, although parts of commercial forest reserves (up to ten percent) can be de-gazetted and reclassified as agricultural lands and converted to industrial plantations. For Indonesian Borneo (Kalimantan), maps of oil palm concession boundaries were obtained from various provincial governments at a 1∶250,000 scale. For South and East Kalimantan provinces, the maps are current as of 2005 [32], [33], [34], [35]. For Central and West Kalimantan provinces, the maps are current as of 2007 and 2008, respectively [36], [37]. If the data were in hard-copy format, they were scanned and digitized in ArcGIS by the Indonesian NGO Living Landscapes Indonesia. For the Malaysian state of Sarawak, maps of oil palm concessions were obtained from AidEnvironment and the Sarawak Dayak Iban Association. Because no official government data of land use are publicly available for Sarawak, these maps were compiled from a range of different sources across different years. In the absence of official up-to-date government maps for Sarawak, we cannot verify the accuracy of the concession data we used in our analysis. For Sabah, there was a dearth of data available regarding spatially explicit concessions. Therefore, to include plantation areas within the analyses we took the “current” plantation extent as a surrogate. To derive this information, we digitised (in ArcGIS 9.3) the extent of plantations throughout Sabah from SPOT 5 2.5 m (2011–2010), SPOT 5 10 m (2009–2007) and Landsat 30 m (2005) satellite images.

#### Industrial tree plantation (ITP) concessions

An ITP concession is a right granted by a government to develop an area of land into an industrial monoculture timber plantation (e.g. *Acacia mangium, Hevea* or *Eucalyptus* spp.). In Indonesia, ITP permits are issued by the Indonesian Ministry of Forestry on lands classified as ‘production forests’. Production forests’ (HPH *Hutan Produksi<300 m asl*. and HPT *Hutan Produksi Terbatas*>300–500 m *asl*) comprise areas allocated for commercial logging, where conversion to another land-use is prohibited. However, the conversion of natural forests to timber plantations is not recognized as deforestation by the United Nations Framework Convention on Climate Change because tree plantations are legally defined as ‘forest’ [38]. In Sarawak and Sabah, ITP permits are issued by the Forestry Department on lands classified as Commercial Forest Reserves (equivalent to Indonesia's production forests). In Sarawak, maps of industrial timber plantation concessions (called Reforestation Licenses, LPF) current as of 2008 were obtained from the Bruno Manser fund report in pdf format [39], scanned and digitized in ArcGIS. For Sabah, maps of Industrial tree plantations (called ITPs) were obtained from digitised Landsat 2000 data (derived from WWF Germany).

#### Logging concessions in natural forests

Companies possessing logging concession licenses have the right to extract natural timber from natural forests. Deforestation (or open clearing) is prohibited in logging concessions, as timber should be extracted in a sustainable manner. For Kalimantan, maps of logging concessions were obtained from the national spatial planning agency (BAPLAN) of the Ministry of Forestry, in 1∶250,000 scale and in digital format. The maps are current as of 2009–2010. For Sabah, a map of ‘production forest’ areas partitioned into forest management units was obtained from the Sabah Forestry Department. For Sarawak, maps of logging concessions for year 1996 were obtained from the Bruno Manser Fund report in pdf format [39], scanned and digitized in ArcGIS. In the absence of official up-to-date government maps for Sarawak, we cannot verify the accuracy of the logging concession map we used in our analysis.

## Results

### Orang-utan distribution model

**Figure 1:**
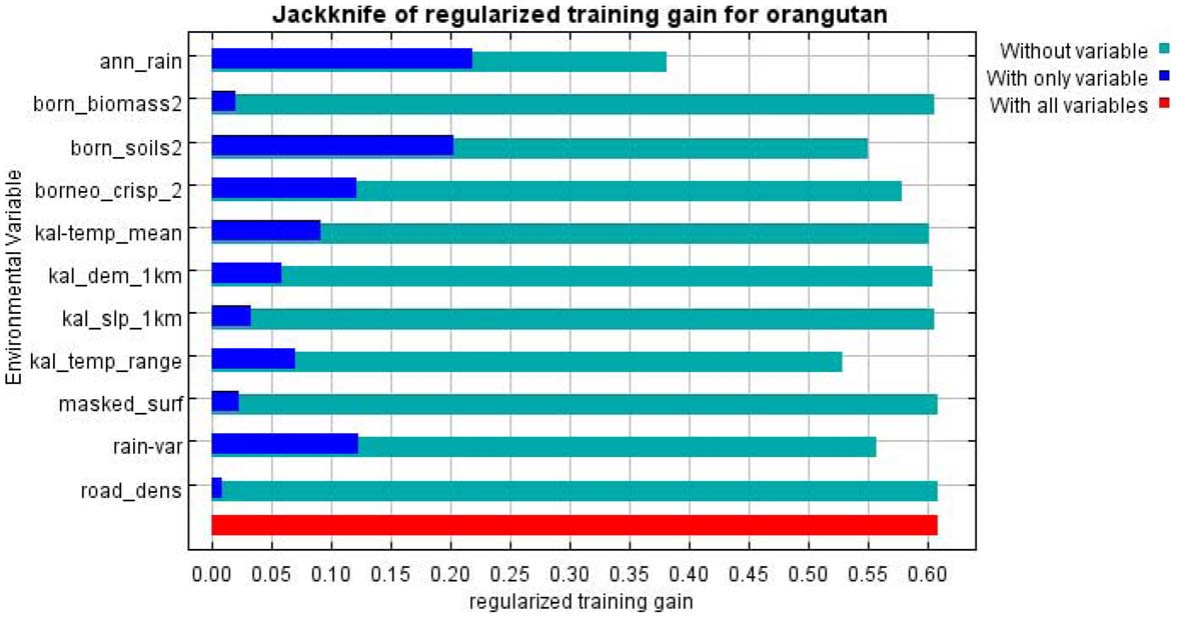
This figure shows the results of the jackknife procedure on the full Maxent model.

**Figure 2:**
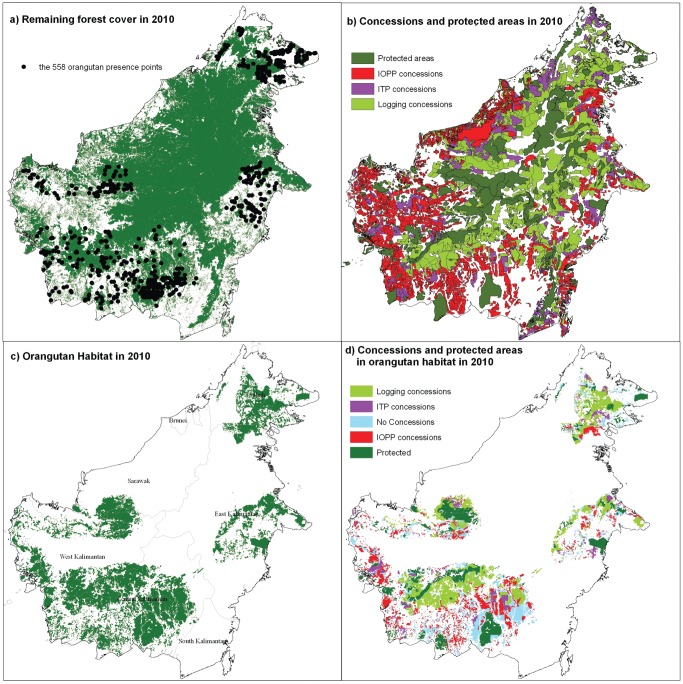
Overview maps of forest cover and orang-utan data points in combination with the orang-utan distribution and land use types. a) Remaining forest cover in 2010. The sample of 558 orang-utan points used to model habitat is shown. b) Concessions and protected areas in 2010, c) The modelled orang-utan spatial distribution. d) Orang-utan distribution and overlap with protected areas and concessions.

The model fit as measured by the mean area under the curve (AUC) of the receiver operating characteristics (ROC) was 0.810 which is considered to be ‘excellent’ [40]. The contextual layer with the highest percentage contribution to the model was annual rainfall (39%), followed by land cover (19%), soil type (15%) and temperature range (14%) (Table 2). Similarly, the jackknife procedure (Figure 1) also indicated annual rainfall by itself contributed more than any other individual variable and that excluding annual rainfall from the model reduced the gain of the model more than the exclusion of any other variable. The latter indicates that annual rainfall contributes to a gain within the model that is not present in any of the other variables. The modelled orang-utan distribution map covers 155,106 km^2^ (21% of Borneo's landmass) (Table 3). It shows four main distinct distribution areas (Figure 2c): a) Sabah and the north-eastern region of East Kalimantan where *P. p. morio* occurs; b) the southern and central East Kalimantan area where *P. p. morio* also occurs; c) the Central Kalimantan and south-western part of West Kalimantan area where *P. p. wurmbii* occurs; and d) the northern part of West Kalimantan and southern part of Sarawak where *P. p. pygmaeus* is found. The largest area of orang-utan distribution is found in Central Kalimantan, followed by West Kalimantan, East Kalimantan, Sabah, Sarawak, and South Kalimantan (Table 3). The area in which orang-utans might occur in South Kalimantan only covers 13 km^2^.

**Table 2:** Contributions of contextual layers to orang-utan Maxent model.

Layer	% contribution (validation model values)
Annual rainfall	39.00 (40.7, 37.7–42.6)
Land cover	19.21 (19.32, 17.41–21.43)
Soil types	15.24 (15.39, 13.30–18.69)
Mean daily temperature range	13.86 (11.58, 10.99–13.27)
Yearly variation in rainfall	7.51 (7.84, 6.73–9.54)
Elevation (DEM)	3.17 (2.64, 2.19–3.23)
Mean daily temperature	0.98 (1.42, 0.91–2.50)
Above-ground carbon stock	0.58 (0.53, 0.18–1.45)
Slope	0.30 (0.39, 0.18–0.69)
Rugosity	0.11 (0.09, 0.02–0.19)
Road density	0.03 (0.09, 0.02–0.48)

Note: validation model values are the mean, min-max values from a 10 fold validation model ran with the same data in Maxent.

**Table 3:** Orang-utan distribution area in protected areas and concessions.

Province State	Total area	in protected areas	in IOPP concessions	in ITP concessions	in logging concessions	Outside concessions	
						in conversion areas	in production areas
West Kalimantan	41,028	12,495	10,525	3,398	5,659	3,200	5,751
South Kalimantan	13	0	0	0	0	13	0
Central Kalimantan	64,673	10,727	14,054	2,259	18,226	8,237	11,170
East Kalimantan	22,695	5,121	4,428	1,597	7,334	2,143	2,072
Sabah	18,632	3,470	No data	1,932	9,317	3,913	0
Sarawak	8,036	2,479	631	746	4,180	0	0
**Borneo**	**155,106**	**34,292 (22%)**	**29,656 (19%)**	**9,899 (6%)**	**44,717 (29%)**	**17,511 (11%)**	**19,031 (13%)**

Note: Areas in this Table are expressed in km^2^. IOPP: industrial Oil Palm Plantation; ITP: Industrial Timber Plantation.

For Sabah data on oil palm plantations concessions were not available.

### Orang-utan distribution in relation to land use

An estimated 29% of the current orang-utan distribution in Borneo is found in natural forests exploited for timber, where logging is allowed but forest conversion is prohibited. A smaller proportion (22%) of orang-utan distribution lies within protected areas where logging and conversion are prohibited (Table 3, Figure 2d). An almost equal percentage (19%) overlaps with largely undeveloped (i.e. still forested) industrial oil palm concessions, and 6% overlaps with largely undeveloped Industrial Tree Plantations. These concessions are still forested but are expected to become converted to plantations in the near future. Finally, an estimated 24% of the orang-utan distribution range occurs outside of protected areas and outside of concessions, with 11% and 13% in “conversion” forests, and in “production” forests, respectively.

## Discussion

### Methodological caveats

This paper provides the latest estimate of the Bornean orang-utan distribution based on the largest geo-referenced survey dataset available. This map makes great improvements upon previous estimates [5], [25]. Nevertheless, within all modelling approaches, commission errors (when a species is mistakenly thought to be present) and omission errors (when a species is mistakenly thought to be absent) are inevitable [41]. We have tried to minimize both kinds of inaccuracies by: (1) allowing the knowledge of orang-utan specialists to facilitate the post-processing of these maps; (2) by running fine-tune iterations of the model to enhance model performance; and, (3) by eliminating areas known to be unsuitable for orang-utans, i.e. areas in which there is no forest anymore (4) as well as areas where orang-utans are naturally found to be absent or only used by transient individuals. This current distribution map has therefore, made significant advancements in understanding the spatial extent of orang-utan distribution and we hope will be pertinent for informing land-use decision making, conservation actions, and associated land use policies.

### Predictor variables

The most important predictor variables for orang-utan distribution in Borneo identified during our analysis were annual rainfall, land cover, soil types and mean daily temperature range. Orang-utans are not predicted to occur in areas of high annual rainfall (eastern part of Sarawak and the northern part of Central Kalimantan for example). High rainfall can influence orang-utan distribution through several indirect processes such as leaching of soils which could lead to less productive forests [42], [43], [44] and/or creating high-cloud cover, which has been shown to lead to a reduction in solar radiation and thereby to lower primary productivity in Borneo [45]. Among the remaining variables, land cover is obviously important for orang-utan distribution since orang-utans in general occur only in areas with some natural tree cover left [17]. The influence of mean daily temperature on orang-utan distribution is also likely to be indirect, although we are not sure about the underlying mechanisms such as potential influences of temperature on thermoregulation that could influence activity patterns, density and distribution [46]. Areas with a very low mean daily temperature do not contain orang-utans and those occur in Brunei (note that we do not suggest that other factors such a rivers, other geographical boundaries or past hunting are not relevant for this gap in the orang-utan distribution) and the far eastern part of Sarawak and the southern part of East Kalimantan. Also, one area of extremely high daily mean temperature variation in Sarawak does not contain orang-utans.

### Contribution of different land-use types to orang-utan conservation

#### Protected areas

No specific study has addressed the relative survival of orang-utans in protected areas compared to other types of land-use type, yet some general patterns can be inferred. Protected areas are the only land-use type where deforestation, logging, and hunting are prohibited – but note that hunting of orang-utans, a protected species, is prohibited everywhere in Borneo. Protected areas are less prone to being degazetted than other land-uses (e.g. logging concessions) and thus may provide more stable long-term habitat. Based on our experiences in both countries (Erik Meijaard, MA, SW, pers obs.) protected areas in Malaysia appear better managed with lower rates of deforestation and forest degradation rates, and fewer incidences of hunting than in Indonesia. In Indonesia, protected areas prevent government-sanctioned deforestation; forest conversion to large-scale agricultural plantations is rare within protected area boundaries [47]. Yet, protected areas appear less effective at preventing smallholder deforestation. Corroborating evidence from throughout the tropics suggests that deforestation persists within protected areas when strong socioeconomic drivers are coupled with insufficient management resources [47], [48], [49], [50], [51], [52]. Protected areas such as the Kutai, Gunung Palung, Sabangau, and Tanjung Puting National Parks in Kalimantan – all home to large orang-utan populations – have been and continue to be affected by illegal logging, encroachments and wildfires [53], [54], [55], [56], [57], [58]. Furthermore, the largest protected areas have generally been established in remote highland areas which are suboptimal for both agricultural purposes and for orang-utans [59], [60]. Additionally governments have disproportionately allocated lowland forests, the preferred habitat of orang-utans, for planned conversion to large scale industrial plantations, transmigration programs, urban and infrastructural development, which all lead to loss, degradation or fragmentation of orang-utan habitat [3], [61]. Thus, although we recognize that protected areas remain an essential aspect of an overall strategy for orang-utan conservation, we argue that the current protected area network is not adequate. Furthermore, it is urgent to re-assess and to re-evaluate the network of protected areas throughout the island to make this network more connected, ecologically resilient, functional, and also to better understand the factors influencing their effectiveness on orang-utan conservation.

#### Logging concessions in natural forests

There is now a large quantity of evidence indicating that selectively logged natural forests can play an important role in the conservation of orang-utan populations [56]. Logging concessions also appear to be equally effective as protected areas in reducing rates of smallholder encroachments [47]. As a result, we are now slowly seeing a change in perception in conservation stakeholders with increased understanding that logging concessions are an important component of maintaining those forest habitats while promoting economic development [62]. However, it is important to recognize that good management is crucial to the effectiveness of these concessions for orang-utan conservation. Concessions where timber is harvested at unsustainable rates tend to have far lower orang-utan densities [16], and other factors such as damage to residual stands and hunting control are also very important. Certification, for example through the principles and criteria of the Forest Stewardship Council (FSC), could guarantee such sustainable management, but participation in certification remains rare in Asian forests because often the costs outweigh the benefits for the companies involved [63]. However, there has recently been progress with the Sabah state government committing to certify all remaining natural and planted forests under FSC by 2015. These positive observations come with an important caveat. Logging concessions are officially designated for sustainable, selective logging, and in theory should remain forested permanently. However, many logging concessions that have been depleted of commercial timber stock, following overexploitation in the past decades have been reclassified for industrial tree plantations in Indonesia and Malaysia and in some cases for oil palm development [64], [65], [66]. Thus, compared to protected areas, logging concessions are more vulnerable to changes in land-use status that might encourage deforestation. Selectively logged natural forest constitutes the highest percentage of the orang-utan distribution of any land-use type (Table 3). Thus, the future of the species largely depends on the strength of the commitments by governments and companies to reduce deforestation and forest degradation rates in logging concessions and to maintain these forests for sustainable timber harvest over the long-term.

#### Industrial tree and oil palm plantation concessions

To increase revenues from predominantly forested landscapes, natural Bornean forests are widely being replaced by oil palm or tree plantations of fast-growing alien species such as *Acacia mangium* and *Eucalyptus* spp. Habitat loss in industrial tree plantations (ITP) and oil palm plantation (IOPP) concessions directly reduces the survival of female orang-utans and their offspring because of limited home range and subsequent inability to move out of deforested areas [67]. Orang-utan males may migrate to remaining forest areas, which will lead to more intense competition among individuals, until those over-crowded areas return to carrying capacity [67]. Therefore, where orang-utan densities are known, the net effect is that area of habitat lost can be used to extrapolate likely losses in orang-utan numbers [3]. Based on the above we estimate that converting a forest area into an industrial plantation will result in the death or displacement of >95% of the orang-utans that originally occurred in the area. One study indicates that monoculture tree plantations may sustain some orang-utans [28], yet it is unclear whether such populations are viable in the long-term. Orang-utans can feed on the bark of acacia trees, often resulting in tree death and concomitant costs to companies [28]. Also, a diet based solely or primarily on bark is likely to have long-term negative physiological impacts on the health of wild populations, although this remains to be studied. As with small-scale agriculture, these tree plantations might provide some resources to orang-utans and could provide essential connectivity between areas of natural forest. Oil palm plantations are the poorest land use type for orang-utans. Recent studies in Sabah indicate that large monoculture oil palm areas devoid of natural tree cover cannot sustain viable orang-utan populations (M. Ancrenaz, unpubl. data). As in other agricultural areas, human-orang-utan conflict is present in areas with oil palm plantations [68]. Despite being a most detrimental land use type in the light of orang-utan conservation, oil palm plantations can nevertheless play a limited (but important) role in connecting natural forest areas, if plantation design incorporates ecological principles of connectivity. For example, in Sabah many orang-utan nests have been found in oil palm plantations that have retained forest corridors along rivers or stepping stones of natural forests within the oil palm matrix (M. Ancrenaz, unpubl. data). Respecting such areas of high conservation value, as for example stipulated by the Round Table for Sustainable Palm Oil [69], should be a good starting point to making oil palm more compatible with the governments' goals to maintain viable populations of threatened orang-utans. However, planning to allow for large-scale orang-utan movement must occur at the landscape level, which requires incorporating a number of different corporations as well as other stakeholders.

#### Areas not under concessions

Forested areas that are not designated as protected areas but are also not currently designated as concessions face three threats. First, they are the targets for provincial and state governments to grant more oil palm concessions on. Second, production forests not under concessions – often because the remaining timber resources are of limited commercial value – could be transferred into Industrial Tree Plantation concessions or for further logging concessions by the Indonesian Ministry of Forestry. Third, many of these forests are being converted for small-scale agriculture by local people. Areas used for small-scale agriculture are generally quite heterogeneous in vegetation structure, and studies in Sumatra [70], [71], [72] and Sabah [73] indicate that these landscapes can in rare cases provide long-term resources to very low density and small localized populations of orang-utans or provide corridors. Human-orang-utan conflicts are, however, frequent and result in many orang-utan deaths, especially when orang-utans get close to villages or feed on crops and in orchards [72]. With traditional shifting cultivation practices in Borneo being replaced by more permanent and intensive monocultural land uses [74]–[75] and human population densities increasing in the region [76], [77], it is expected that these small-scale agricultural landscapes will become increasingly less suitable for orang-utan survival. If hunting, however, can be reduced significantly, these landscapes might play an important role in providing connectivity between forested areas, as shown in the Kinabatangan floodplain in Sabah [78].

## Conclusions

Our analysis indicates that approximately 78% of the distribution of Bornean orang-utans falls outside of protected areas, and the land-use types in which orang-utans most frequently occur are logging concessions (29% of distribution) and protected areas (22% of distribution) which retain good natural forest cover. We sketch two possible scenarios for the future of orang-utans in Borneo. In the first, business-as-usual scenario, areas under: a) oil palm plantation concession (19% of current orang-utan distribution), b) industrial timber plantation concessions (6% of current orang-utan distribution), and c) outside concessions (24% of current orang-utan distribution) would become deforested. Under this scenario at best only 51% of the current orang-utan distribution (in protected areas and logging concessions) would remain and 49% of the current distribution would largely be lost. This scenario is conservative, as it assumes that protected areas will maintain the current level of forest cover, and that all logging concessions will be maintained, both of which are unlikely. In addition, this analysis does not factor in the impacts on orang-utan populations from hunting, which has been shown to be a substantial threat in Kalimantan in particular [7]. We also did not consider the impact of mining concessions, many of which are being developed in the forested interior of Borneo and potentially add to large-scale forest degradation and clearing, especially in extensive coal basins in East Kalimantan. In a second possible scenario, all forests that are outside concessions but are classified under ‘production’ status would be maintained as natural forests areas for logging, rather than relegated as concessions for industrial tree plantations or converted for other uses (e.g., agriculture). As these forests currently contain 13% of the orang-utan distribution (Table 3), approximately 64% of the current orang-utan distribution would remain if all such forests, protected areas and logging concessions maintain orang-utans. Similarly, with scenario one, this is likely to be too optimistic and the true value would likely be lower than 64%. If the Indonesian government is to adhere to its commitment to stabilize all wild orang-utan remaining in 2007 by 2017 [14], and the Sabah government to its commitment of maintaining viable orang-utan populations [15], some major changes to current land-use patterns need to be made. First and foremost, expansion of oil palm and tree plantations in remaining orang-utan habitats should be halted as it infringes national laws on species protection. Further growth of the plantation sector should be achieved through increasing yields in existing plantations and expansion of new plantation into areas that have already been deforested. Figure 2 shows the orang-utan distribution presently allocated for plantation development, and we strongly recommend that all these areas are placed under a conversion moratorium and subsequently reallocated to land uses that are compatible with species conservation goals (logging concession or protected area). Growth of the plantation sector as targeted by both the Indonesian and Malaysian governments should be achieved through increased yields in existing plantations and expansion of new plantation into areas that have already been deforested.

To ensure long-term survival of orang-utans, a masterplan at the landscape level is needed that will consider all remaining viable populations as well as all the different land uses that are active within the orang-utan's range. Such a master plan should clarify which possible land uses and managements are allowed in the landscape and provide new standardized strategic conservation policies (e.g., a policy for logging concession management in orang-utan areas). In Indonesia the National Spatial Plan provides an opportunity to create such a masterplan, but a first step in this is that all discrepancies between the various government spatial planning plans need to be solved and integrated into one national spatial plan that is adhered to by all government levels [6]. Much work has been done on the types of management that would be compatible with conservation objectives, as exemplified by criteria and indicators under RSPO and FSC [79]. Policies and regulation should determine the roles and responsibilities of different people and organizations involved on how to manage land and forests and how to abate threats to orang-utans, such as hunting. Overall, better land use planning is needed in which costs and benefits of particular land use choices are carefully considered. Furthermore, even when governments have this information it does not necessarily result in actions promoting orang-utan conservation. For example, the provincial government of Aceh in northern Sumatra recently granted the right to develop an oil palm plantation on peat swamp forest area, deemed ‘empty’ of orang-utans by the authorities [80], even though this area had previously been identified and publicized as key orang-utan habitat by conservationists [5], [6], [14], [81], [82]. Just before this paper went to print, this concession was revoked, however, after a massive national international media campaign and several court cases. The above example demonstrates the need for enhanced communication and collaboration between scientists, conservation practitioners, policy makers, industry and other key stakeholders. This is vital for developing informed land-use planning, particularly considering that not all key wildlife habitats can become strictly protected. Such a process should make much better use of values of ecosystem services of forests such as water provision, flood control, carbon sequestration, and sources of livelihood for rural communities. Presently land use planning is more driven by vested interests and direct and immediate economic gains, rather than by approaches that take into consideration social equity and environmental sustainability [6], [31]. Both the Malaysian and Indonesian governments have committed to the long-term maintenance of natural capital, but this requires the use of scenarios that integrate the need for both economic growth and environmental and social sustainability when making land use decisions. The science to assist this process is becoming increasingly advanced and efficient, and is available for use by governments and other planning bodies [6], [83]. Still, the general mindset appears to be that environmental conservation and economic development are mutually exclusive, as expressed by the East Kalimantan governor who stated that in the choice between people and orang-utans, the former should take precedence [1].
